# Controlled Hypothermic Storage for Lung Preservation: Leaving the Ice Age Behind

**DOI:** 10.3389/ti.2024.12601

**Published:** 2024-04-17

**Authors:** Ismail Cenik, Jan Van Slambrouck, An-Lies Provoost, Annalisa Barbarossa, Cedric Vanluyten, Caroline Boelhouwer, Bart M. Vanaudenaerde, Robin Vos, Jacques Pirenne, Dirk E. Van Raemdonck, Laurens J. Ceulemans

**Affiliations:** ^1^ Department of Thoracic Surgery, University Hospitals Leuven, Leuven, Belgium; ^2^ Laboratory of Respiratory Diseases and Thoracic Surgery (BREATHE), Department of Chronic Diseases and Metabolism, KU Leuven, Leuven, Belgium; ^3^ Department of Pulmonology, University Hospitals Leuven, Leuven, Belgium; ^4^ Abdominal Transplant Surgery, University Hospitals Leuven, Leuven, Belgium; ^5^ Immunology and Transplantation, Department of Microbiology, KU Leuven, Leuven, Belgium

**Keywords:** controlled hypothermic storage, lung preservation, lung transplantation, mitochondrial health, static ice storage

## Abstract

Controlled hypothermic storage (CHS) is a recent advance in lung transplantation (LTx) allowing preservation at temperatures higher than those achieved with traditional ice storage. The mechanisms explaining the benefits of CHS compared to conventional static ice storage (SIS) remain unclear and clinical data on safety and feasibility of lung CHS are limited. Therefore, we aimed to provide a focus review on animal experiments, molecular mechanisms, CHS devices, current clinical experience, and potential future benefits of CHS. Rabbit, canine and porcine experiments showed superior lung physiology after prolonged storage at 10°C vs. ≤4°C. In recent molecular analyses of lung CHS, better protection of mitochondrial health and higher levels of antioxidative metabolites were observed. The acquired insights into the underlying mechanisms and development of CHS devices allowed clinical application and research using CHS for lung preservation. The initial findings are promising; however, further data collection and analysis are required to draw more robust conclusions. Extended lung preservation with CHS may provide benefits to both recipients and healthcare personnel. Reduced time pressure between procurement and transplantation introduces flexibility allowing better decision-making and overnight bridging by delaying transplantation to daytime without compromising outcome.

## Introduction

Preservation of organs remains a key area for research in transplantation medicine. Several strategies to reduce organ injury during preservation have been explored. Over the past decades, organ-specific preservation solutions and *ex-vivo* perfusion platforms have been developed [1–4]. Currently, the standard of care for preservation remains static ice storage (SIS). Traditionally, temperature in this setting has been estimated to be around 4°C [1, 5]. However, actual temperature with SIS might be lower and result in freezing injury due to the organ contact with ice [6]. On a cellular level, the low temperatures reached with SIS decrease metabolic demand, but has also been associated with progressive mitochondrial dysfunction [7]. Interestingly, lungs are privileged because the oxygen repleted air in the expanded alveoli continues to support aerobic metabolism during preservation [8]. Recently, it has been shown that controlled hypothermic storage (CHS) of lungs at 10°C better maintains mitochondrial health during preservation [9, 10]. Therefore, the optimal temperature for hypothermic lung preservation might be found by balancing aerobic metabolism and oxygen consumption while preventing cellular exhaustion [7, 11, 12]. With SIS, cold ischemia time (CIT) for lungs is advised to be kept as short as possible to minimize cellular injury [1, 13]. The limited preservation time forces teams to perform transplant procedures at night, and restraints long-distance procurement. Overnight transplantation is associated with worse post-transplant outcomes, possibly due to limited resources and expertise, and sleep-deprived personnel [14, 15]. Lung preservation with CHS at a higher temperature has the potential to safely extend preservation time and thereby overcome logistical challenges [9, 16]. In this article we aim to provide a focus review of preclinical research, the cellular mechanisms, and the clinical experience explaining the benefits of CHS in the setting of lung transplantation (LTx).

## Preclinical Data on Controlled Hypothermic Lung Storage

**FIGURE 1:**
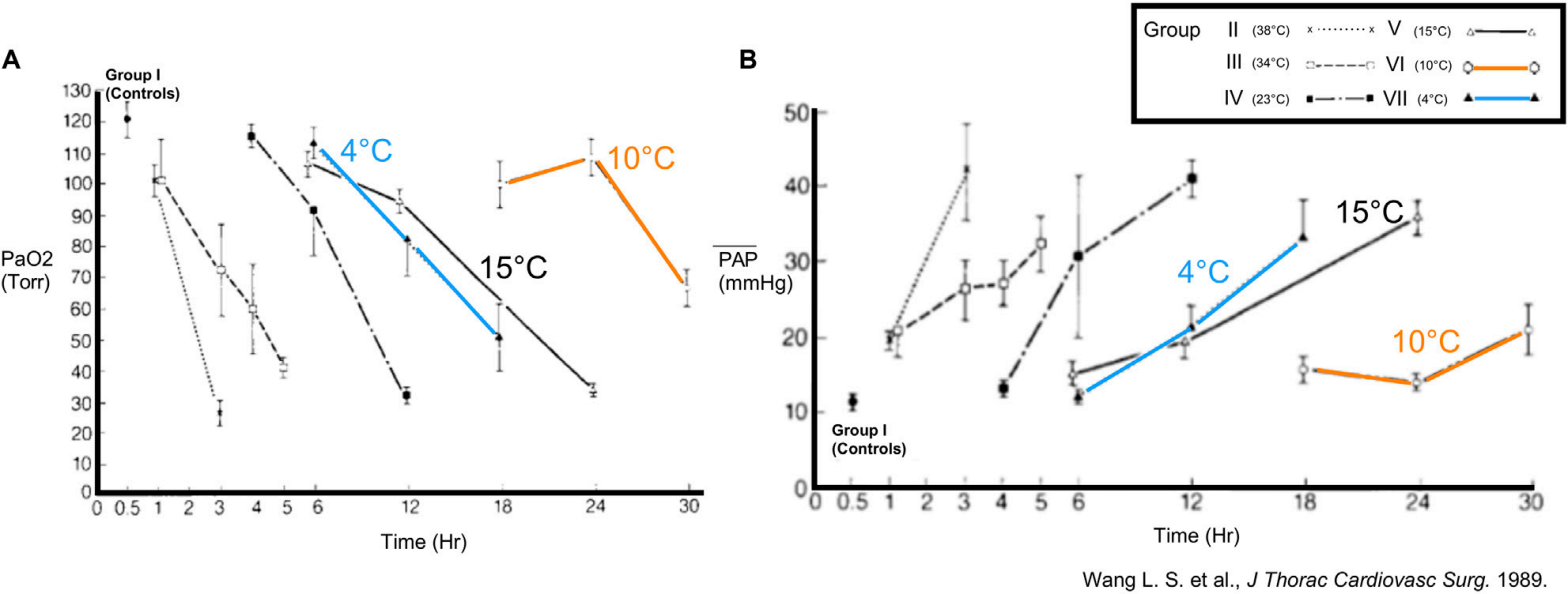
Findings in a rabbit model of *ex-vivo* lung preservation and reperfusion (Toronto, 1989). Physiology in lungs inflated with room air was assessed after different intervals of ischemia and preservation temperatures. Groups with 4°C, 10°C and 15°C are highlighted. **(A)** Partial arterial oxygen pressure (PaO_2_) and **(B)** mean pulmonary artery pressure (mPAP) were measured after 10 min of reperfusion. Reprinted from The Journal of Thoracic and Cardiovascular Surgery, Vol. 93(3), Liang-Shun Wang, Koichi Yoshikawa, Shinichiro Miyoshi, Kembu Nakamoto, Chia-Ming Hsieh, Fumio Yamazaki, Paulo F. Guerreiro Cardoso, Hans-Joachim Schaefers G, Julio Brito, Shafique H. Keshavjee, Alexander Patterson, Joel D. Cooper, The effect of ischemic time and temperature on lung preservation in a simple ex vivo rabbit model used for functional assessment, pp. 333-342, Copyright (1989), with permission from Elsevier.

Over the past decades, concerns regarding optimal temperature for lung preservation have been raised due to cold-induced lung injury provoked by SIS. In 1989, Wang and colleagues (Toronto, Canada) developed an *ex-vivo* rabbit lung preservation model with the aim to accurately assess post-ischemic lung function [17]. For lungs with different CIT and preservation temperatures (4°C, 10°C, 15°C, 23°C, 34°C, 38°C), they assessed post-ischemic functional variables like partial pressure of arterial oxygen (PaO_2_), pulmonary artery pressure (PAP) and oxygen uptake (Figure 1). Of note, the 4°C group was performed in a temperature-controlled room, and not by immediate ice contact. They hypothesized that lower temperature would improve post-ischemic lung function. However, after 12 h CIT followed by 10 min reperfusion, lungs preserved at 10°C were functionally superior to those preserved at 4°C. The 10°C group tolerated CIT periods of 18–24 h with PaO_2_ and PAP similar to 30 min preservation at 4°C. The extension of CIT to 30 h deteriorated gas exchange (Figure 1). These observations raised questions regarding optimal lung preservation temperature and led to a revision of the dogma advocating cold storage of lungs at 4°C.

**FIGURE 2:**
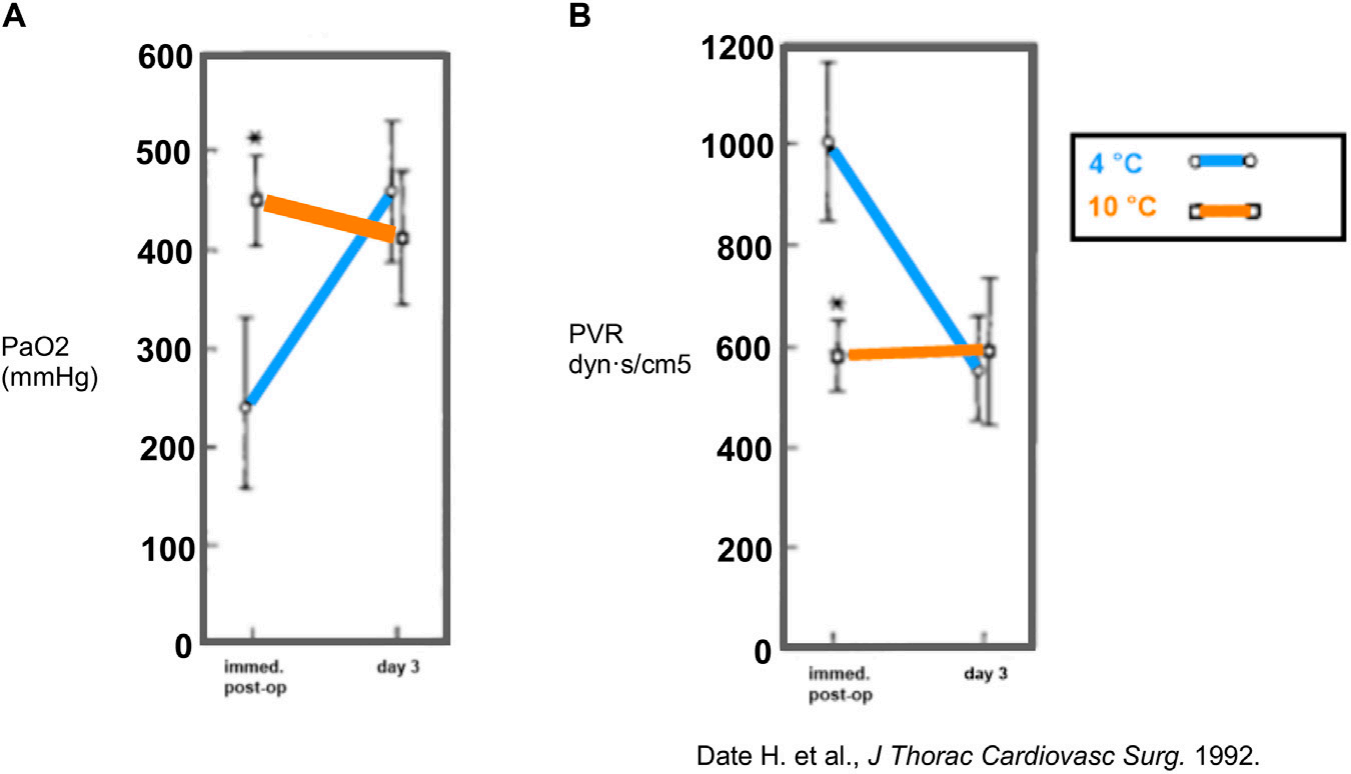
Findings in a canine model of orthotopic left-lung transplantation (St. Louis, 1992). The left lung was inflated with room air and preserved at 10°C or 4°C for 18 h. During occlusion of the contralateral pulmonary artery, partial arterial oxygen pressure (PaO_2_) **(A)** and pulmonary vascular resistance (PVR) **(B)** were measured immediately post-LTx (n = 6/group) and at day 3 post-LTx (n = 4/group). Mean values are shown and * indicates *p* < 0.05. Reprinted from The Journal of Thoracic and Cardiovascular Surgery , Vol. 103(4), Hiroshi Date, Oriane Lima, Akihide Matsumura, HiroharuTsuji, D. André d'Avignon, Joel D. Cooper, In a canine model, lung preservation at 10° C is superior to that at 4° C. A comparison of two preservation temperatures on lung function and on adenosine triphosphate level measured by phosphorus 31-nuclearmagnetic resonance, pp. 773-780, Copyright (1992), with permission from Elsevier.

In 1992, Date et al. (St. Louis, Missouri) set up a canine model of orthotopic left-LTx to compare pulmonary function in lungs filled with room air after 18 h of preservation at 10°C vs. 4°C [18]. Furthermore, they conducted a pulmonary metabolic study evaluating adenosine triphosphate (ATP) levels. Functionally, gas exchange and perfusion were superior in the 10°C group with higher PaO_2_ and lower pulmonary vascular resistance (PVR) immediately post-LTx. No important difference in PaO_2_ or PVR was observed on day 3 (Figure 2). In addition, ATP levels remained stable during the 18 h preservation at 10°C and 4°C. Impaired lung physiology at 4°C preservation could hence not be explained by cellular ATP depletion. Therefore, it was suggested that sodium/potassium (Na+/K*+*) ATPase might be impaired at 4°C, leading to intracellular Na + accumulation and cellular swelling. The St. Louis researchers (1992) concluded that optimal lung preservation temperature is near 10°C [18].

Also in 1992, Nakamoto et al. (Kagawa, Japan) preserved rabbit lungs for 18 h at predetermined temperatures ranging from 4°C to 15°C [19]. Physiology was assessed with PAP, PaO_2_ and wet-to-dry (W/D) ratio as a measure for edema. Pulmonary vasculature was assessed with perfusion of blood containing indocyanine green (ICG) and histopathological evaluation was performed using colloidal carbon black perfusion.

**FIGURE 3:**
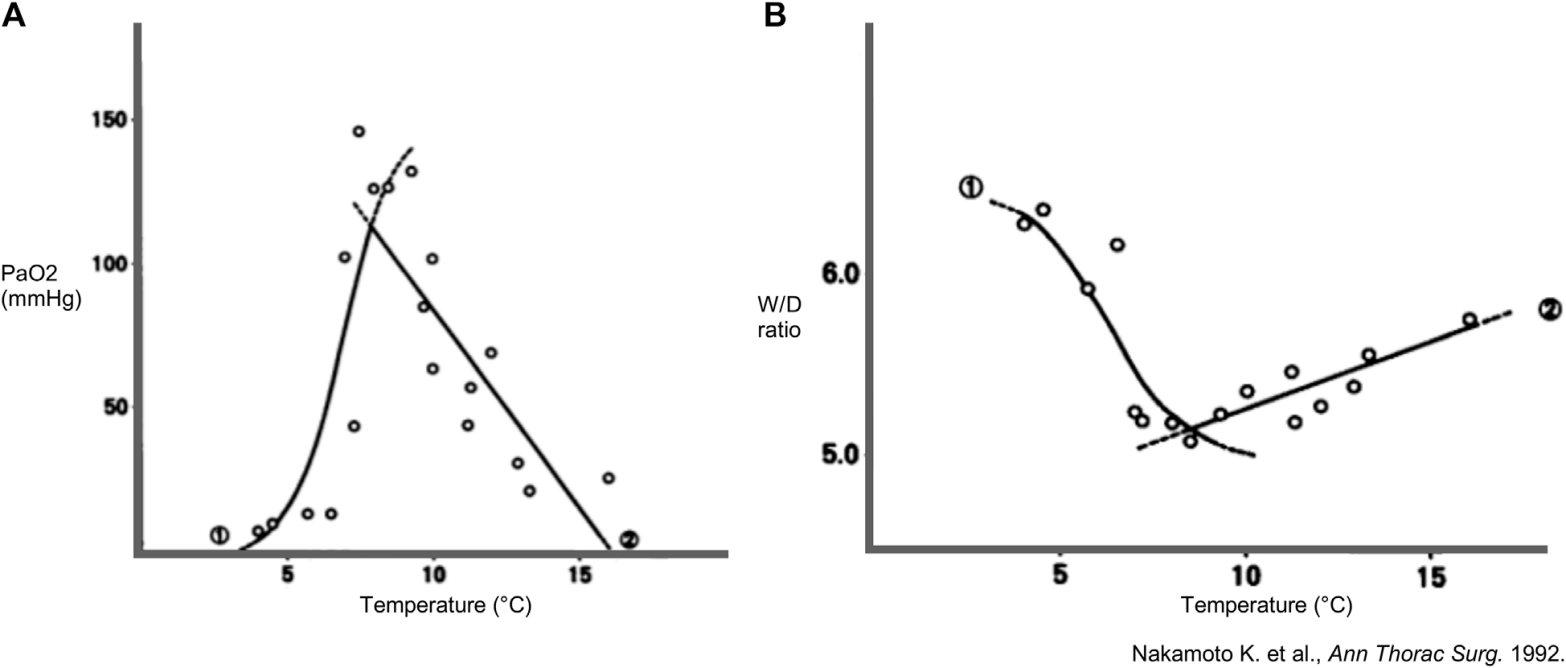
Findings in a rabbit model of lung preservation and *ex-vivo* reperfusion. Regression lines and curves during high-flow perfusion with ventilation and lung preservation temperature. Every dot represents a measurement. **(A)** Partial arterial oxygen pressure (PaO_2_) (n = 19) and **(B)** wet-to-dry weight (W/D) ratio (n = 16) of lungs inflated with room air and preserved for 18 h in function of different preservation temperatures. Mathematically projected significant regression equations 1 and 2 intersect at 7.9°C for PaO_2_ and at 8.4°C for W/D ratio. Reprinted from The Annals of Thoracic Surgery, Vol. 53(1), Kembu Nakamoto, Masazumi Maeda, KiyohideTaniguchi, Noriyuki Tsubota, Yasunaru Kawashima, A study on optimal temperature for isolated lung preservation, pp. 101-108, Copyright (1992), with permission from Elsevier.

Based on mathematical regressions of W/D and PaO_2_ (Figure 3), optimal temperature for lung preservation was found between 8°C and 9°C. At 8°C, superior physiology was observed, and microvasculature was intact in contrast to other groups. Each fitted sigmoid curve for W/D and PaO_2_ showed an inflection point at 6°C–7°C, which seems to be a critical temperature for vascular obstruction. After 4°C preservation, carbon colloid particles were absent in capillaries and massive alveolar hemorrhage with arteriolar obstruction was seen, suggesting impaired physiology was related to vascular obstruction. However, at 15°C, carbon colloid particles were present, and centrilobular alveolar hemorrhage was observed, suggesting deterioration at 15°C was due to increased injury to the alveolocapillary membrane rather than vascular obstruction [19].

Other extended preservation experiments were performed in canine left LTx models by Keshavjee et al. (Toronto, 1989), Mayer et al. (Toronto, 1991) and a porcine left LTx model by Steen et al. (Lund, 1993) [20–23]. However, further translation of CHS to clinical practice was considered a major challenge due to lack of insights into the underlying mechanisms and logistical demands.

**FIGURE 4:**
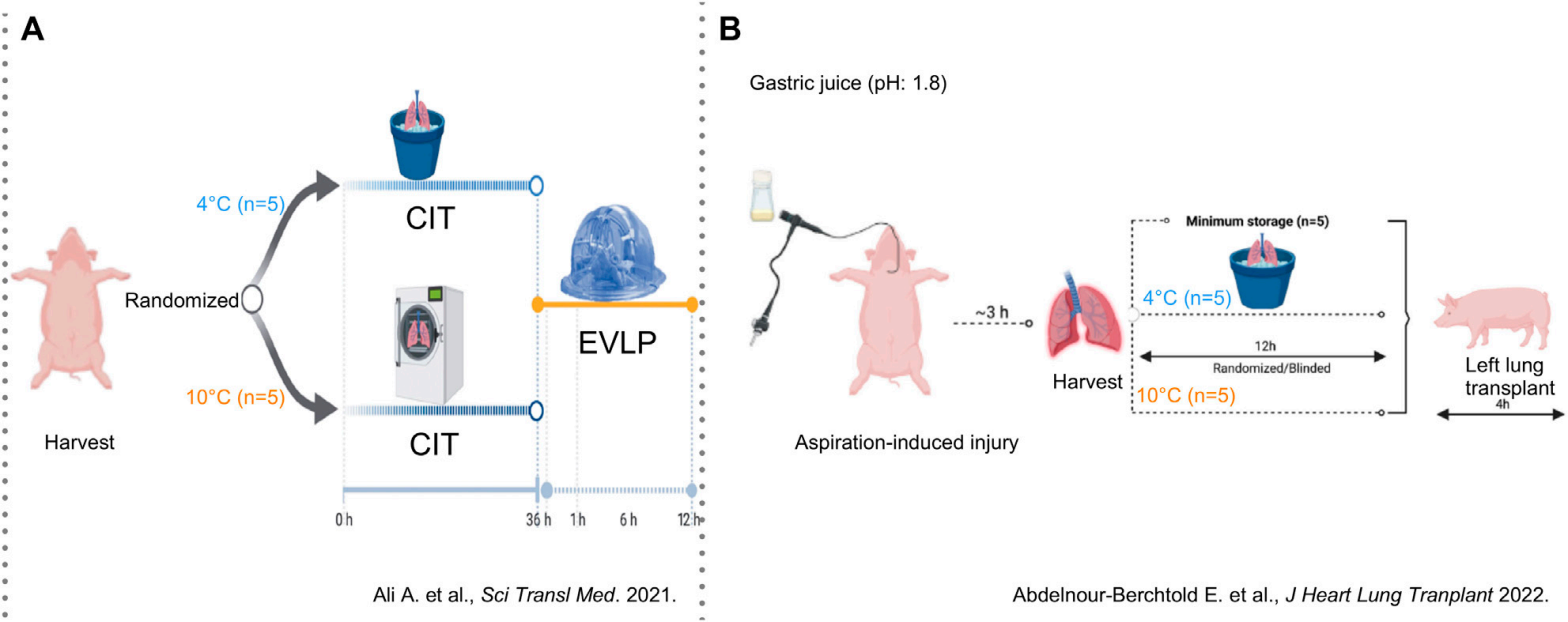
Findings in a porcine model of lung preservation followed by *ex-vivo* perfusion (Toronto, 2021) or orthotopic left lung transplantation (Toronto, 2022) **(A)** Lungs were inflated with inspired oxygen (FiO_2_) of 50% and preserved for 36 h in a cooler at 4°C or in a cooler at 10°C (n = 5/group) followed by 12 h of normothermic *ex-vivo* lung perfusion (EVLP). Lung physiology and molecular patterns in lung tissue and EVLP were assessed. From Aadil Ali et al., Static lung storage at 10°C maintains mitochondrial health and preserves donor organ function. Sci. Transl. Med.13,eabf7601(2021). DOI:10.1126/scitranslmed.abf7601. Reprinted with permission from AAAS. **(B)** Lungs were injured with instillation of gastric juice and procured 3 h later with 50% FiO2. Preservation was done for 1 h (n = 5) or for 12 h on ice (n = 5) or at 10°C in a cooler (n = 5). Lung graft physiology and molecular patterns of injury were assessed after orthotopic left lung transplantation followed by 4 h of reperfusion. Reprinted from The Journal of Heart and Lung Transplantation, Vol. 41(12), Etienne Abdelnour-Berchtold, Aadil Ali, CristinaBaciu, Erika L. Beroncal, Aizhou Wang, OliviaHough, Mitsuaki Kawashima, Manyin Chen, YuZhang, Mingyao Liu, Tom Waddell, Ana C. Andreazza, ShafKeshavjee, Marcelo Cypel, Evaluation of 10°C as the optimal storage temperature for aspiration-injured donor lungs in a large animal transplant model, pp. 1679-1688, Copyright (2022), with permission from Elsevier.

Three decades later, in 2021, the concept of 10°C preservation with the intention of better preserving lung physiology and metabolism re-emerged in Toronto where Ali, Cypel and colleagues developed a porcine model of lung preservation followed by *ex-vivo* lung perfusion (EVLP) (Figure 4A). Pig lungs were randomized to storage at 4°C (walk-in cooler) or 10°C (thermoelectric cooler) for 36 h followed by 12 h normothermic EVLP [9].

**FIGURE 5:**
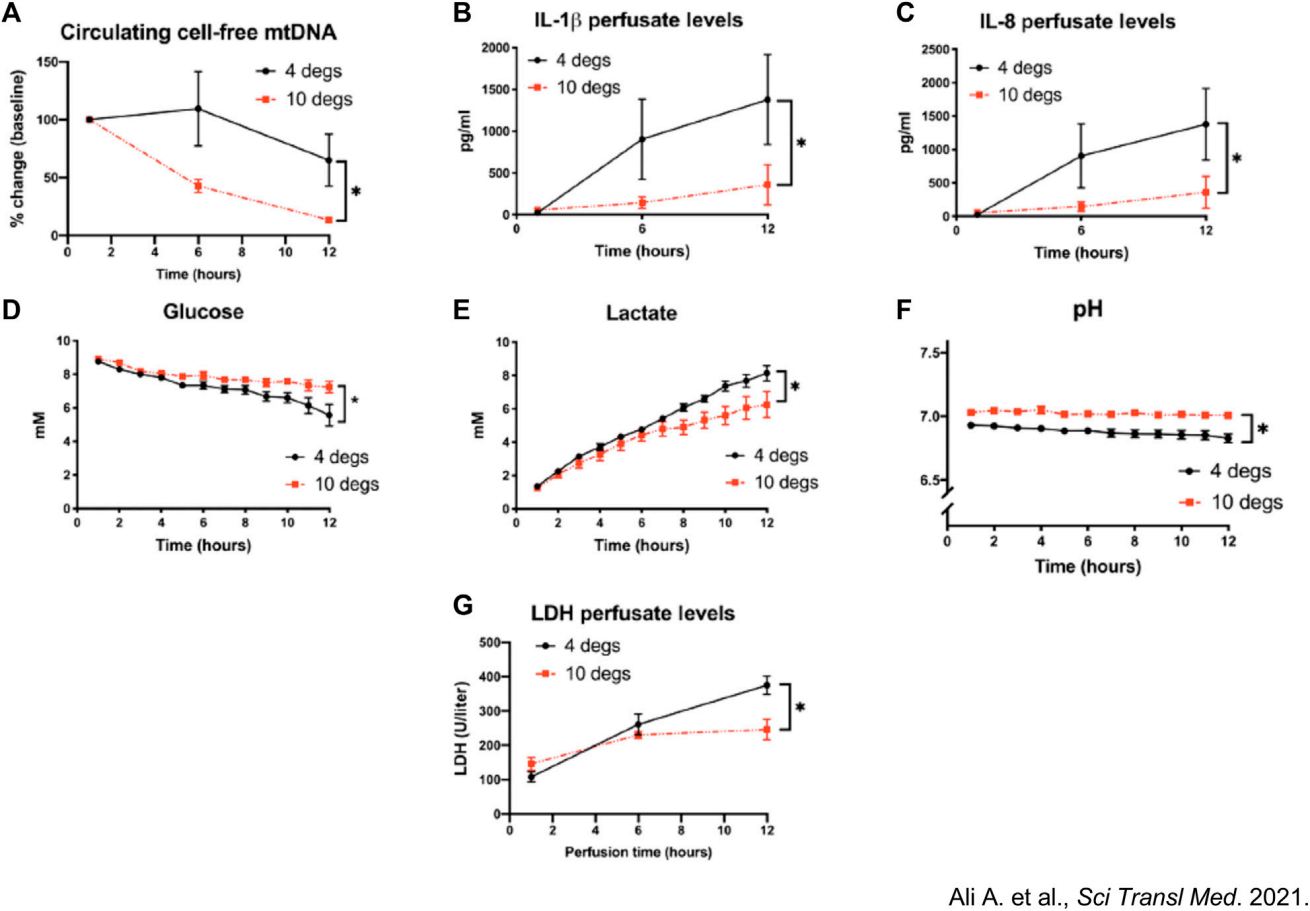
Assessment of mitochondrial health between pig lungs stored at 10°C versus 4°C. Pig lungs were filled with 50% inspired oxygen (FiO_2_) and preserved at 10°C vs. 4°C for 36 h. **(A)** Circulating cell-free mitochondrial DNA (mtDNA) extracted from normothermic *ex vivo* lung perfusion (EVLP) perfusate and quantified using qPCR during lung perfusion. Fold changes from baseline concentrations (1-h perfusion). **(B–G)** Perfusate **(B)** Interleukin 1β (IL-1β) **(C)** IL-8 levels, **(D)** glucose, **(E)** lactate and **(F)** pH. **(G)** LDH activity within EVLP perfusate, a measure of cellular necrosis. *p < 0.05, two-way ANOVA; data expressed as means ± SEM. From Aadil Ali et al., Static lung storage at 10°C maintains mitochondrial health and preserves donor organ function. Sci. Transl. Med.13, eabf7601(2021). DOI:10.1126/scitranslmed.abf7601. Reprinted with permission from AAAS.

CHS at 10°C resulted in higher compliance, better oxygenation, and less edema. Furthermore, CHS was associated with upregulated expression of cytoprotective anti-oxidative metabolites (itaconate, glutamine, N-acetyl glutamine) as well as decreased perfusate protein levels of pro-inflammatory cytokines and cell-free mitochondrial DNA (mtDNA) (Figure 5) [9].

In a porcine model of orthotopic left LTx, Abdelnour-Berchtold et al. (Toronto, 2022) studied potential benefits of 10°C storage in marginal lungs injured by 5 mL gastric juice (Figure 4B) [10]. Three hours after instillation, lungs were procured and randomized to 10°C or ice for 12 h followed by LTx. As control group, five injured lungs were placed on ice for 1 h followed by LTx.

Four hours post-Tx, 10°C preservation resulted in superior selective left-lung PaO_2_, compliance, and histopathological evidence of reduced acute lung injury. Based on metabolomic analysis, they found no metabolic activity for SIS. However, 10°C preservation showed active metabolism and higher levels of anti-oxidative metabolites (glutathione, ascorbate). Tissue levels of interleukin-1β were significantly lower for 10°C preservation. Compared to controls, free mtDNA was expressed less at 10°C.

These findings suggest that intentional delay of LTx by using CHS might trigger cell protective mechanisms and result in better post-LTx outcome.

## Ice Is Not 4°C

**FIGURE 6:**
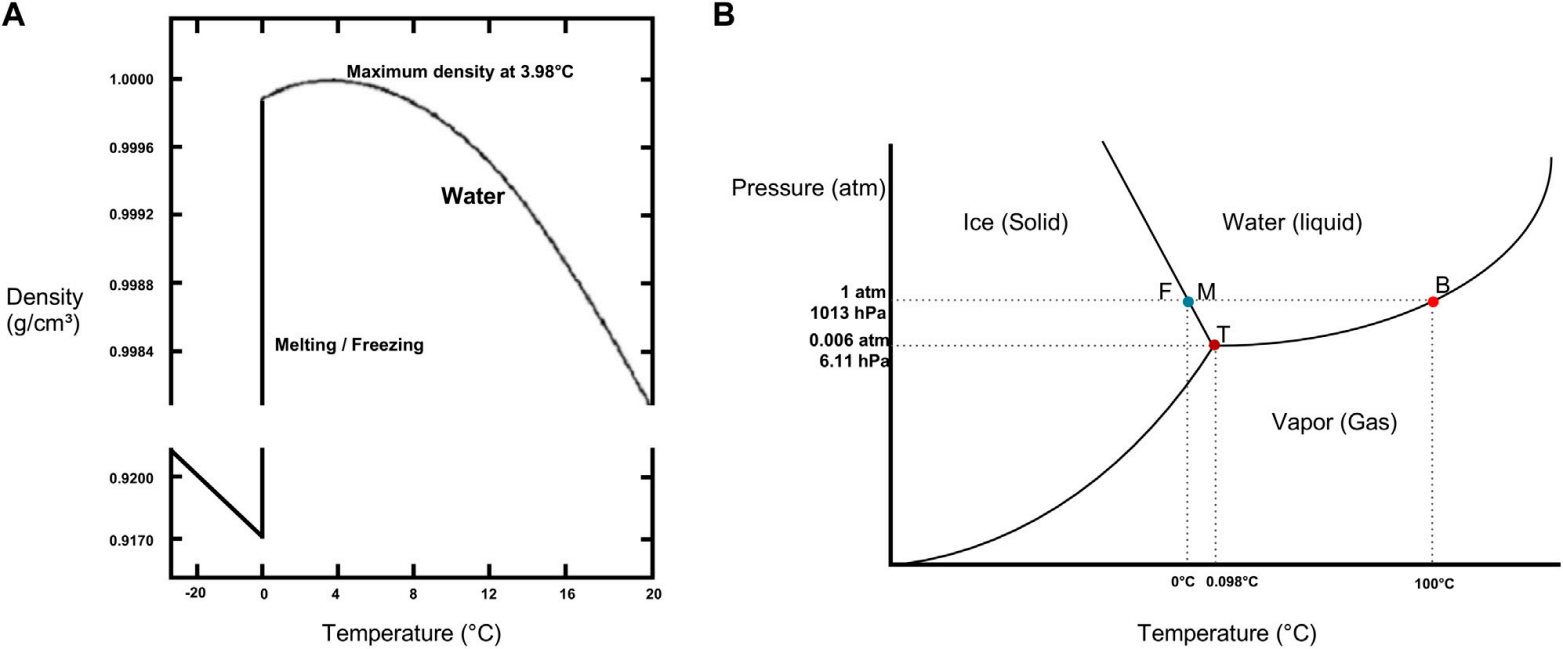
Physical properties of water **(A)** Density of water/ice as a function of temperature. Note maximum density of water at 4°C and freezing (F) or melting (M) point of water at 0°C. Data from the CRC Handbook of Chemistry and Physics [24]. **(B)** Phase diagram of water illustrating triple point (T), boiling point (B) and freezing (F) or melting (M) point in function of pressure (P) and temperature. For 1,013 hPa corresponding to the atmospheric pressure (atm), freezing point of water is at 0°C and boiling point of water is at 100°C. Data obtained from CRC Handbook of Chemistry and Physics [24].

The International Society for Heart and Lung Transplantation (ISHLT) consensus statement on organ preservation advises to avoid contact of organs with ice to limit cold-induced cellular injury ([6]). In transplantation, a misconception exists regarding temperatures reached by SIS, wherein it is commonly assumed that organs are maintained at 4°C. However, no temperature measurements of donor lungs during SIS were available. This misconception of 4°C might refer to the temperature at which water reaches its maximum density in a soluble state. The actual freezing temperature of water at atmospheric pressure (1013 hPa) is 0°C, at which water is present in the form of solid ice (Figure 6). Interesting to note is that slushed ice used in organ preservation is derived from 0,9%NaCl physiologic saline with a freezing point below 0°C, approximating −0,59°C. Furthermore, a saline cooling model by Robicsek et al. noted temperature drops in clinical setting to −7,1°C and osmolality differences between the center and periphery of the solid ice [25].

**FIGURE 7:**
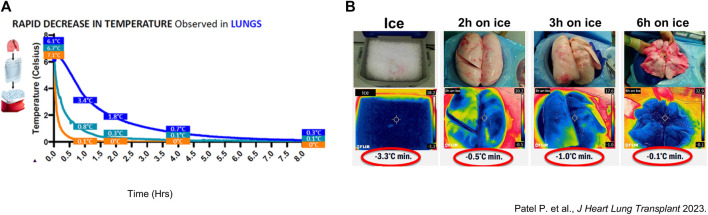
Thermodynamic assessment of porcine and clinical lung temperature during static ice storage **(A)** Average temperature of porcine lungs (n = 3) preserved on ice (blue). Measurements were performed with a probe in the left lung lower lobe. Another probe measured the preservation solution (Perfadex) in contact with the lungs (cyan) and saline solution (n = 3) (orange). Temperatures were measured every 30 s. **(B)** Thermal images of ice used for standard static ice storage (SIS), and surface temperature of clinical lungs preserved for 2-, 3- and 6-hours.

In the earlier described canine LTx model, temperature dropped below 1°C after 2 h of SIS [18]. In a recent preliminary study of porcine lung preservation thermodynamics, Patel et al. showed that preservation and tissue temperature reached around 0°C after 3 h of SIS (Figure 7) [26].

**FIGURE 8:**
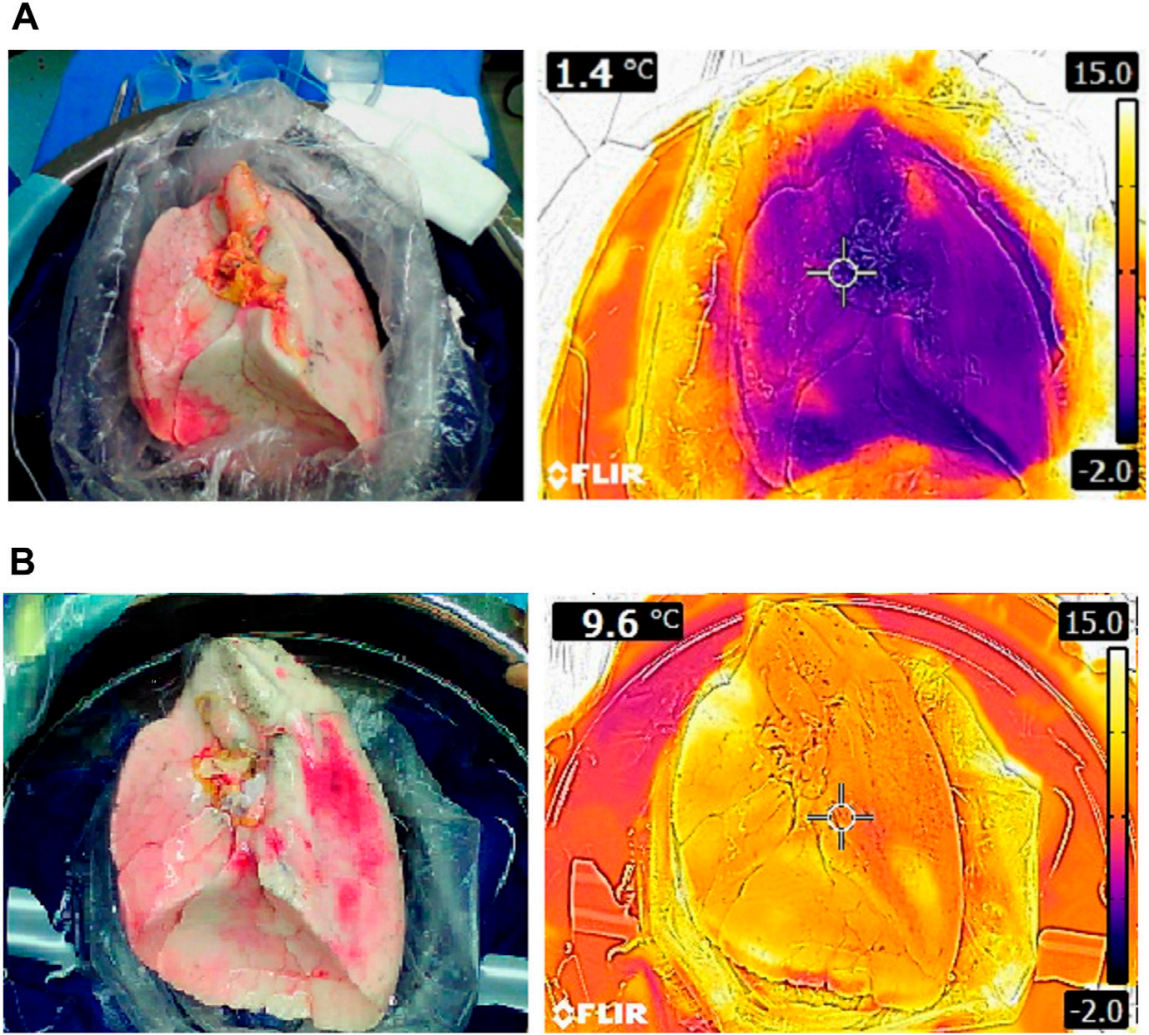
Thermal images of left lungs preserved with static ice storage versus controlled hypothermic storage in clinical lung transplantation: **(A)** Right lung after 228 min of SIS (ice cooler) and **(B)** right lung after 276 min of CHS (LUNGguard).

In a clinical study at our center (Leuven, Belgium) donor lung temperatures were measured after preservation with SIS on ice [27]. Median parenchymal surface temperature measured with a thermal camera (Figure 8) and core temperature measured with a probe (3 mm) wedged in a lower lobe bronchus was close to 0°C with SIS.

## Cold-Induced Cellular Injury and the Mechanisms by Which Controlled Hypothermic Storage Protects Cellular Viability

### Static Ice Storage Causes Cold-Induced Cellular Freezing Injury

**FIGURE 9:**
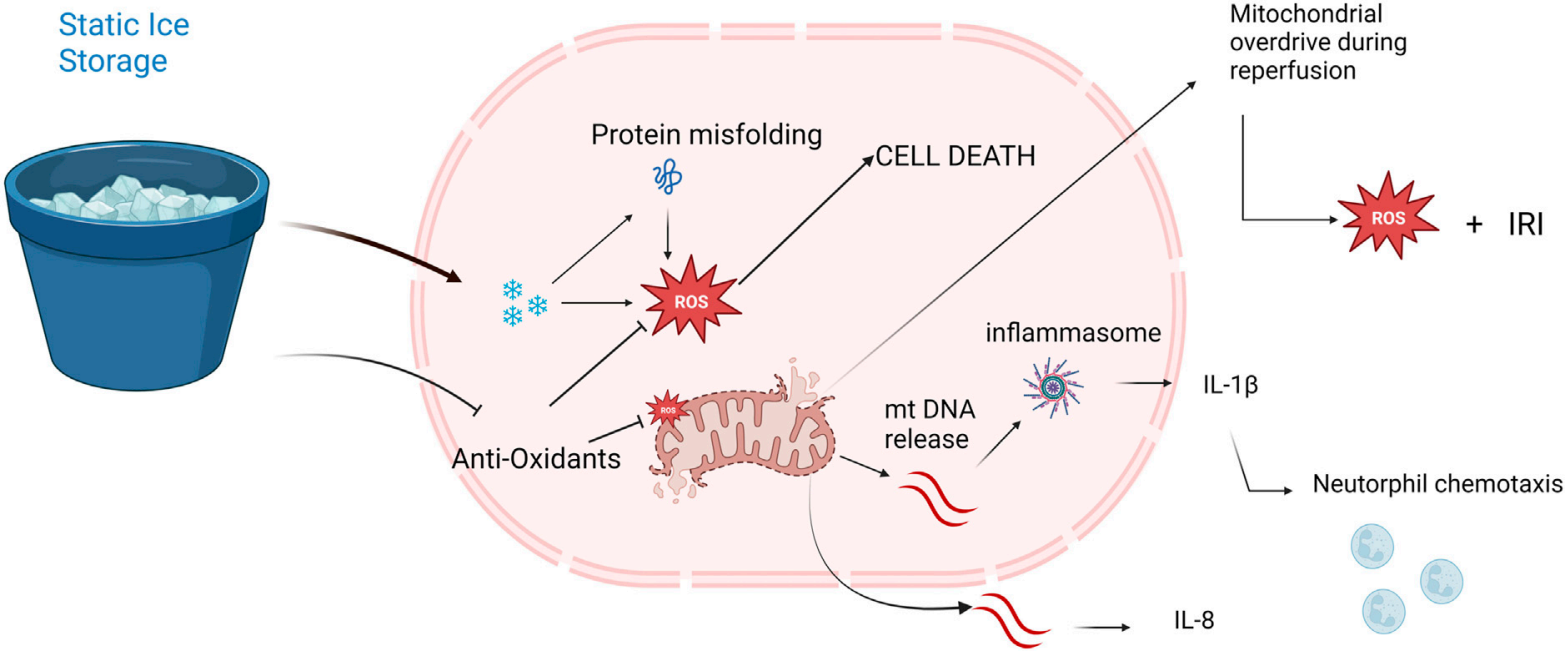
Potential mechanisms of freezing injury and mitochondrial dysfunction provoked by static ice storage. Freezing injury is mediated via formation of miniscule ice crystals disturbing electrolyte-and homeostatic balances. Near freezing temperatures destabilize hydrogen bonds resulting in misfolding of globular proteins. Halted metabolism decreases the levels of anti-oxidative metabolites like glutathione and ascorbate. Damaged mitochondria release mitochondrial DNA (mtDNA) which triggers the inflammasome pathway resulting in neutrophil chemotaxis and increased inflammatory cytokine release (e.g., interleukin-8, interleukin-1β). At reperfusion, depletion of mitochondria results in a state of mitochondrial overdrive during which excessive amounts of reactive oxygen species (ROS) are produced causing extensive ischemia reperfusion injury (IRI).

Cellular structure and metabolic function are altered at low temperatures (Figure 9). Cold-induced protein denaturation is caused by changes in hydrophobic amino-acid interactions at near-freezing temperatures and are responsible for loss of stability and misfolding of globular structural proteins [28]. In tissues exposed to temperatures around 0°C, formation of ice crystals causes electrolyte and osmotic imbalances leading to mitochondrial swelling and dysfunction [29].

Cellular survival depends on ATP metabolism and the main source for ATP is aerobic metabolism in the mitochondria. Furthermore, mitochondrial injury and subsequent cell death results in the release of mtDNA which triggers inflammatory pathways [9, 30]. Mitochondrial dysfunction is also associated with increased levels of reactive oxygen species (ROS) which further propagate mitochondrial disintegration and oxidative stress [31]. Taken together, safeguarding mitochondrial function is essential to maintain organ quality during preservation.

### The Role of the Na+/K + ATPase During Static Ice Storage and Controlled Hypothermic Storage

**FIGURE 10:**
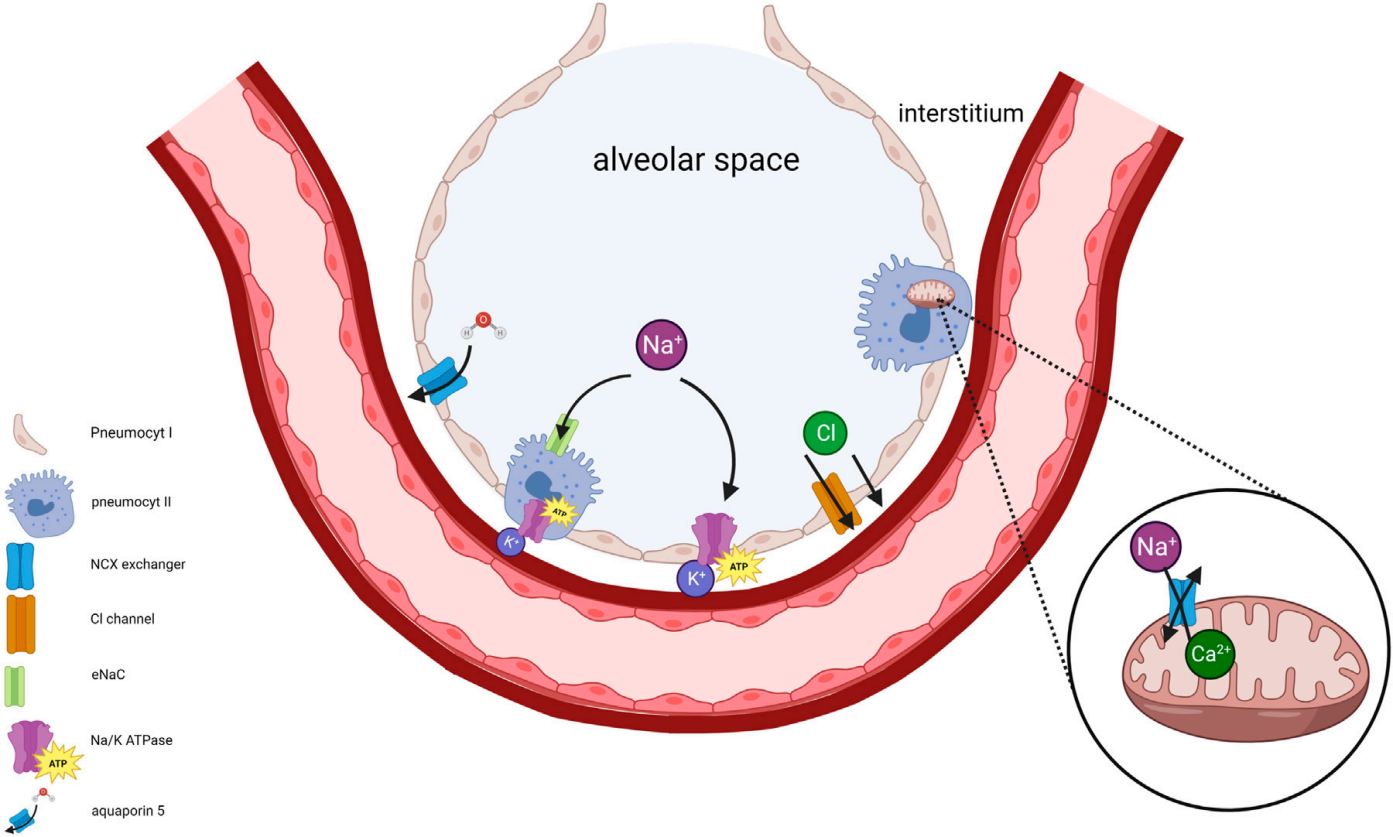
The significance of maintaining Na^+^/K^+^ ATPase activity in pneumocytes and mitochondria is illustrated. Disruption of intracellular Na^+^ concentration, resulting from impaired Na^+^/K^+^ ATPase, disturbs membrane potentials. This disruption triggers an elevation of cytosolic Ca^+2^ concentration followed by inhibition of mitochondrial Ca^+2^ efflux primarily due to impaired Na^+^/Ca^+2^ exchanger (NCX) activity. Consequently, intramitochondrial retention of Ca^+2^ occurs accompanied by mitochondrial swelling. Type I and II pneumocytes provide alveolar fluid clearance (AFC) through vectorial ion transport, generated by an osmotic gradient towards the interstitium. Na^+^ enters the pneumocyte via apical epithelial Na^+^ channels, subsequently exiting through the basolateral Na^+^/K^+^ ATPase. Cl^−^ moves towards interstitium via the cystic fibrosis transmembrane conductance regulator (CFTR). Water follows along the osmotic gradient.

The Na+/K + ATPase maintains cell membrane potential. Dysfunction of Na+/K + ATPases results in extramitochondrial accumulation of Na+ and Ca+2 followed by impairment of the mitochondrial Na+/Ca+2 pump (NCX). ATP depletion and impaired Na+/K + ATPase function therefore cause accumulation of intramitochondrial Ca+2 resulting in mitochondrial swelling and dysfunction [32]. The intracellular accumulation of Ca+2 activates proteases which leads to an irreversibly conversion of xanthine dehydrogenase to xanthine oxidase. Xanthine oxidase and increased hypoxanthine is responsible for ROS formation during anoxic ischemia reperfusion injury (IRI) [33]. Alveolar fluid clearance (AFC) via type I and II pneumocytes depends on an osmotic gradient towards the interstitium created by Na + transport across the apical membrane through epithelial sodium channelss and basolateral Na+/K + ATPase (Figure 10).

In rabbit and canine models of lung preservation, it was shown that aerobic mitochondrial metabolism is sustained during preservation. In contrast to other solid organs, the lung is indeed privileged by its supply of oxygen in the air-filled alveoli. Interestingly, the analyses showed that ATP levels were equal during 1°C/4°C and 10°C preservation [8, 18]. Therefore, improved organ preservation with CHS cannot be explained by improved Na+/K + ATPase function. In the porcine model of lung preservation, comparative analysis of Na+/K + ATPase activity during preservation showed no significant difference between 4°C and 10°C [9]. However, Na+/K + ATPase is essential to maintain organ quality. During an experiment where Na+/K + ATPase was inhibited by ouabain during 10°C preservation and subsequent EVLP, lung physiology was significantly impaired [9].

### Controlled Hypothermic Storage Favors Aerobic Mitochondrial Metabolism

It must be recognized that the mechanisms of ROS formation are different in lung tissue in comparison to other solid organs due to the presence of oxygen during ischemia. In other solid organs like the liver or heart, ischemia during preservation is coupled to anoxia, a process called anoxic ischemia. Depletion of oxygen in the alveoli impairs aerobic cellular respiration in the mitochondria. ATP synthesis is consequently shifted towards anaerobic glycolysis and phosphocreatine [8].

**FIGURE 11:**
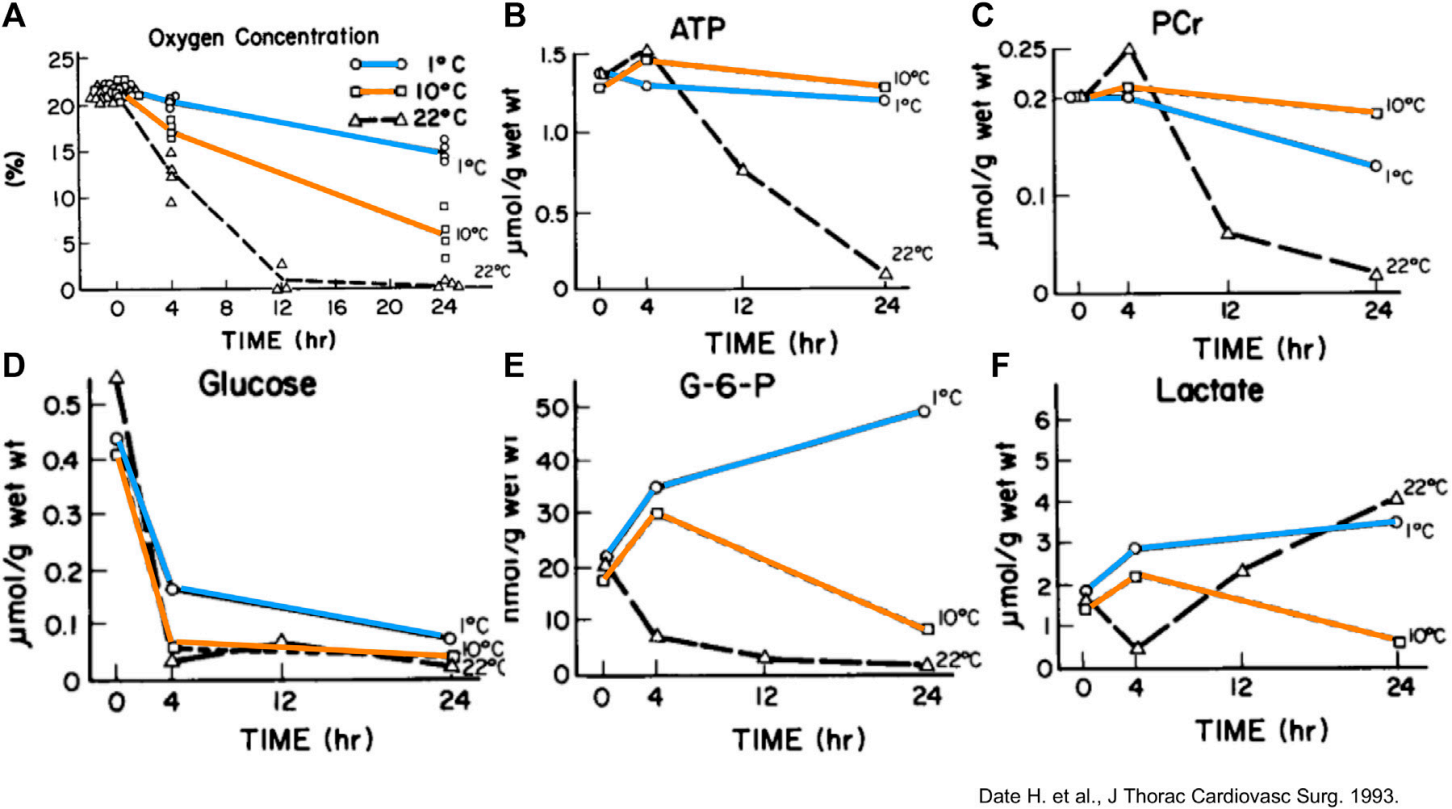
Measurements and/or means of gas concentration and metabolic parameters in rabbit lungs in function of time (Hr) preserved at different temperatures. **(A)** Oxygen concentration in the airway. Each point represents one experiment. **(B)** Mean Adenosine triphosphate (ATP). **(C)** Mean phosphocreatine (PCr). **(D)** Mean glucose. **(E)** Mean glucose-6-phosphate (G-6-P). **(F)** Mean lactate. Reprinted from The Journal of Thoracic and Cardiovascular Surgery, Vol.105(3), Hiroshi Date, Akihide Matsumura, Jill K. Manchester, Joshua M. Cooper, Oliver H. Lowry, Joel D. Cooper, Changes in alveolar oxygen and carbon dioxide concentration and oxygen consumption during lung preservation The maintenance of aerobic metabolism during lung preservation, pp. 492-501, Copyright (1993), with permission from Elsevier.

In a rabbit model of 24 h lung preservation, metabolic characteristics were compared at 1°C, 10°C and 22°C (Figure 11) [8]. Oxygen consumption and metabolic need increased with higher temperatures. Energy stores were depleted fastest at 22°C. 1°C preservation resulted in minimal ATP consumption with preserved glucose and glucose-6-phosphate levels over time. However, 1°C preservation beyond 4 h led to increased lactate levels in the 1°C group suggesting cell death. Conversely, lactate levels decreased over time during 10°C preservation [8, 34]. Furthermore, ATP and phosphocreatine levels were highest after 24 h of 10°C preservation.

### Controlled Hypothermic Storage Favors Anti-oxidative Metabolism

In the porcine model of lung preservation after gastric juice instillation, a shift towards anti-oxidative metabolism was found for 10°C preservation [10]. Higher lung tissue levels of glutathione and ascorbate were detected after 10°C compared SIS, suggesting that CHS favors anti-oxidative metabolism. Glutathione and ascorbate are scavenger molecules that neutralize oxygen radicals and are consumed at ischemia-reperfusion injury (IRI) [35]. Higher levels of anti-oxidative metabolites reduce cellular oxidative stress and in turn better preserve mitochondrial health [9, 10, 36].

**FIGURE 12:**
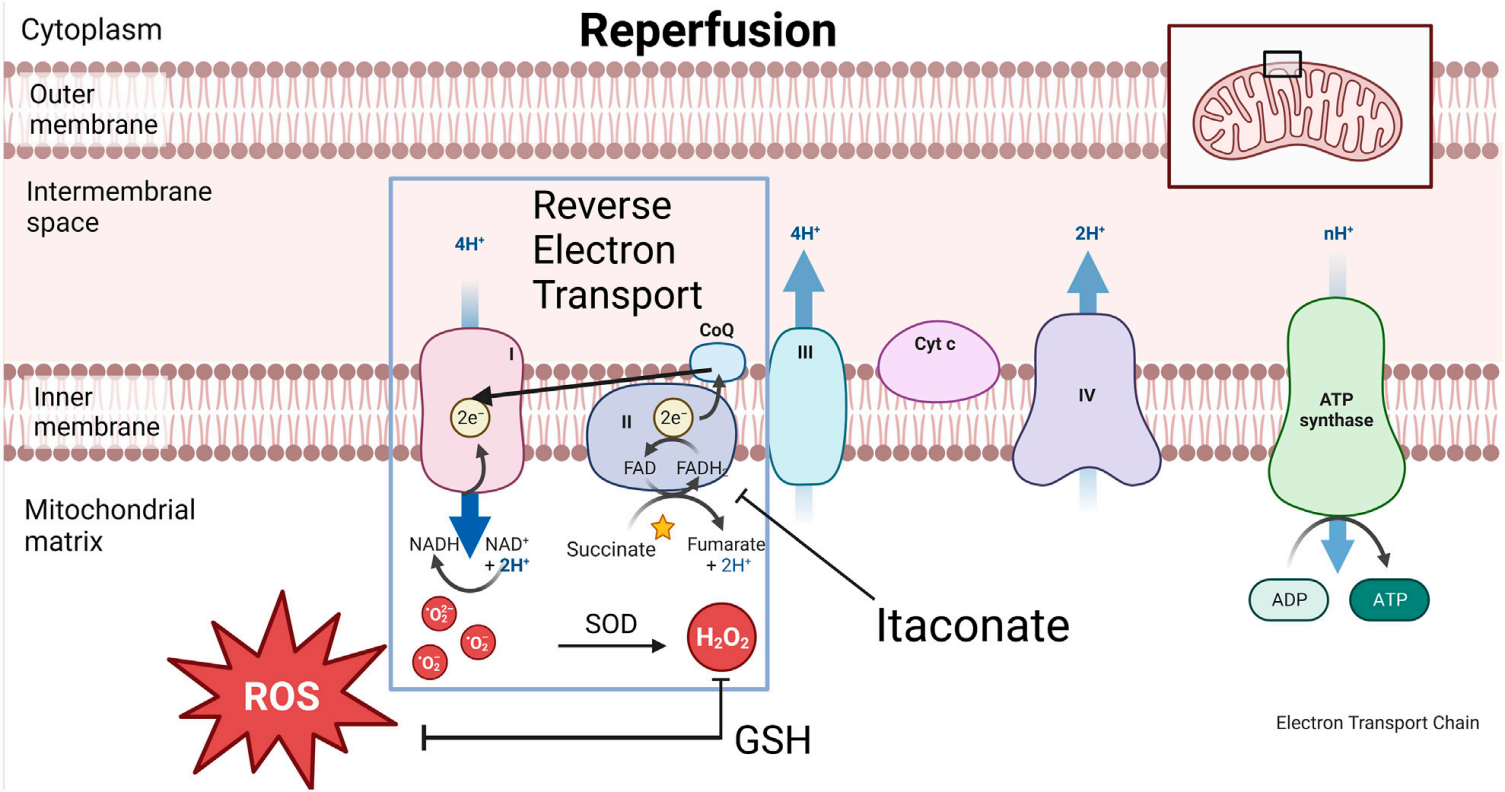
Graphical representation of cytoplasm, mitochondrial inner-and outer membrane and electron transport chain (ETC) during reperfusion. During reperfusion, the electron transport chain suddenly awakens with increasing temperature. Reactive oxygen species (ROS) formation via reverse electron transport (RET) is associated with ischemia reperfusion injury (IRI). During this process, Complex II or succinate dehydrogenase (SDH) in the ETC is overwhelmed by the amount of electron passage presented by succinate. Electrons slip away from the CoQ pool back to complex I, a process called RET during which oxygen radicals are formed. This process is inhibited by itaconate which enables a gradual awakening of the ETC and reduced ROS formation. Superoxide dismutase (SOD) transforms oxygen radicals into H_2_O_2_ which the clearance of partially depends on the antioxidative metabolite glutathione (GSH).

Metabolic profiling after 36 h preservation of porcine lungs showed increased tissue levels of glutamine, n-acetyl glutamine and itaconate at 10°C vs. 4°C [9]. Accumulation of succinate during ischemia acts as a potential electron store which in turn drives superoxide formation with the introduction of oxygen during reperfusion (Figure 12) [37–39]. Itaconate inhibits succinate dehydrogenase (Complex II) and decreases succinate-derived formation of ROS at reperfusion and can therefore explain the association between higher itaconate levels in lung tissue after 10°C CHS with reduced lung injury during EVLP after 36 h preservation [9, 38].

Taken together, CHS of oxygen repleted lungs appears to achieve the right balance of preventing freezing injury and cellular exhaustion, ideally between 8°C and 10°C where cellular metabolism is preserved producing anti-oxidative metabolites, while maintaining efficient oxygen consumption.

## Hypothermic Storage Devices for Donor Lung Preservation

### myTEMP 65HC Incubator (Benchmark Scientific)

**FIGURE 13:**
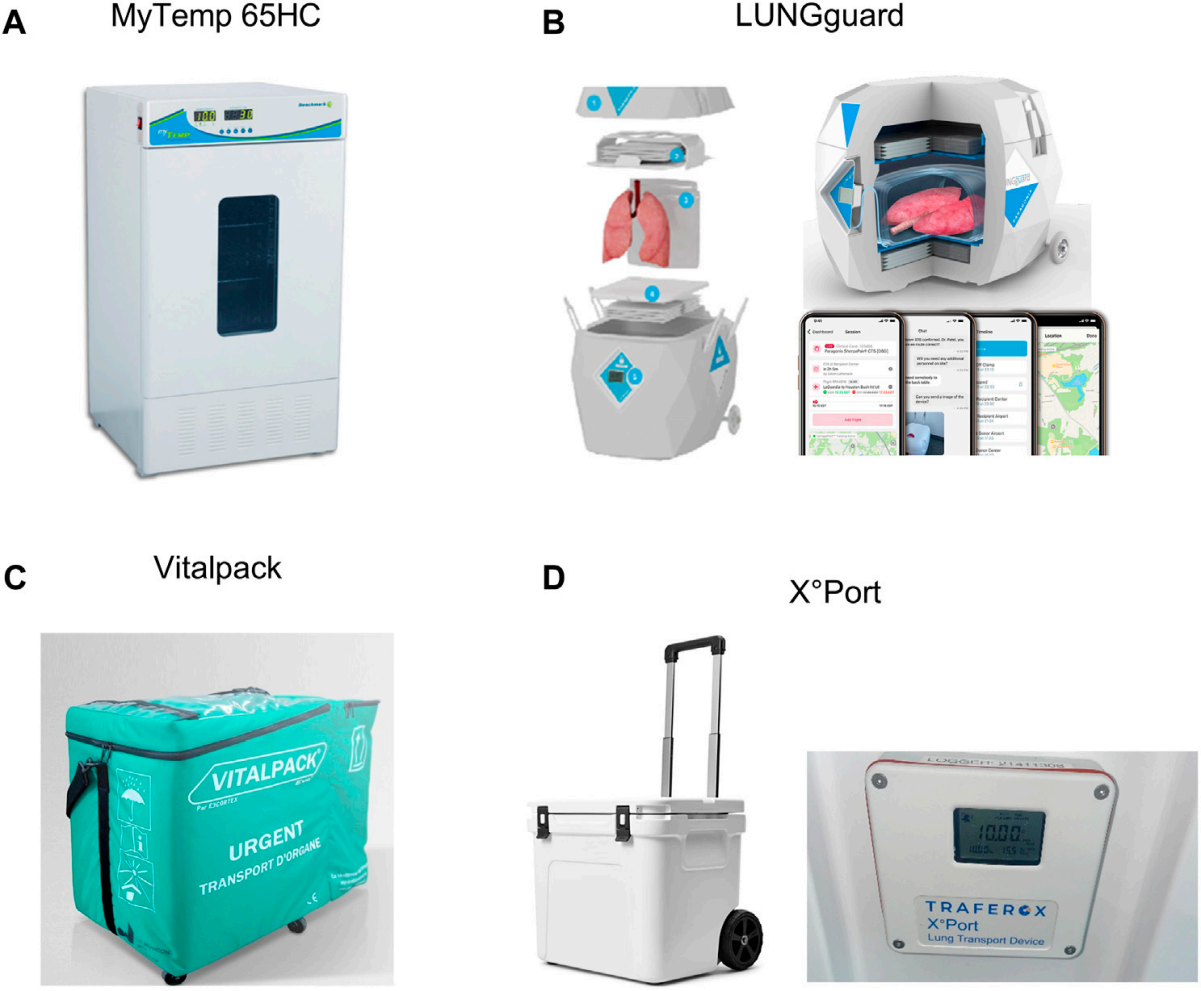
Images of hpothermic preservation devices used for/or available for controlled hypothermic storage (CHS) of donor lungs. **(A)** The myTEMP 65HC Incubator, Benchmark Scientific. **(B)** LUNGguard, Paragonix. **(C)** VITALPACK^®^ EVO, E3 CORTEX. **(D)** X °Port Lung Transport Device (Traferox Technologies Inc.).

An overview of the different controlled hypothermic storage devices is summarized under Table 1. The myTEMP 65HC Incubator allows storage at an accurate and stable temperature ranging from 0°C to 60°C. A large viewing window is available for visual monitoring (Figure 13A). This device meets the electrical and technical specifications for use in an operating theatre (cf. infra). It is not portable and does not allow CHS during organ transport.

**TABLE 1:** Overview of hypopothermic preservation devices used for/or available for controlled hypothermic storage (CHS) of donor lungs. (A) The myTEMP 65HC Incubator (Benchmark Scientific) used in preclincial CHS studies, proof-of-concept study and prospective non-randomized multicenter trial initiated by the Toronto group. (B) LUNGguard PARAGONIX being used for the ongoing GUARDIAN-LUNG regitsry. (C) VITALPACK^®^ EVO, no data available for the lungs. (D) X°Port Lung Transport Device (Traferox Technologies Inc.) being used in an ongoing prospective randomized controlled multicenter trial initiated by the Toronto group.

	MyTemp 65HC incubator	LUNGguard	Vitalpack Evo	X°Port lung transport device
Cooling source	Electrical	Sherpa cooling	Eutectic plates	Gel packs
Preparation	Custom temperature by user	48 h: −20°C storage	24 h: −18°C storage	48 h on 4°C
Temperature range	10°C	4°C–8°C	2°C–8°C	10°C
Time between temperature range	Continuous	40 h	40 h	36 h
FDA	X	✓	X	X
CE-Mark	X	✓	✓	x
Monitoring	Temperature	Temperature and location	VITALTRACK	Temperature and location
			Temperature and location	
Data on lung storage	Preclinical and clinical	Ongoing clinical	No data available	Ongoing clinical
	- proof-of-concept	- Guardian lung registry		- Prospective multicenter randomized controlled trial
	- prospective multicenter non-randomized trial			

### LUNGguard™ (Paragonix)

LUNGguard (Paragonix, Boston, MA, United States), the first commercially available portable device was first used in clinics in 2021 (Duke Hospital, Durham, United States) and focuses primarily on preventing ice-contact related injury during lung CHS [40]. It received Food and Drug Administration (FDA) clearance and a Conformitéee Européenne (CE) mark (Figure 13B). LUNGguard is designed for CHS at a temperature ranging from 4°C to 8°C. Sherpa Cooling technology with phase-changing material must be cooled at −20°C for 48 h and provides CHS up to 40 h. A smartphone application connected to a logger and thermometer in the LUNGguard allows remote real-time monitoring of location and storage temperature. Also, CHS devices exist for heart (SherpaPak) and liver (LIVERguard).

Moreover, recently, BAROguard received FDA-clearance and its first clinical utilization has been reported [41]. In addition to the features of LUNGguard, BAROguard automatically controls airway pressure of donor lungs during preservation within the recommended range, which is relevant during air transport to avoid pressure-related injury.

### VITALPACK EVO (E3 CORTEX)

The Class IIa Medical Device VITALPACK^®^ EVO is an organ transport container that complies with the Directive 93/42/EEC, used for packaging, transport and preservation of organs (Figure 13C). VITALPACK provides storage of all organs at 2°C–8°C. The source of cooling consists of four gel packs which are to be frozen at −18°C for at least 24 h and placed at room temperature for 30 min before inserted into the box. VITALPACK ensures a start temperature inside the box of 8°C, decreasing to >2°C with a preservation duration of minimal 40 h (tests provided by company on website) [42]. It is designed and guaranteed for 10 uses. VITALTRACK is an extra service in the form of a device that allows location and temperature monitoring. No data has been reported on lung preservation. VITALPACK is standard use for all organ preservation in France since 2017, and has recently been implemented in Switzerland (personal communication E3 CORTEX).

### X°Port Lung Transport Device (Traferox Technologies Inc.)

X°Port Lung Transport Device (Traferox Technologies Inc.) has been developed by the Toronto Lung Transplant Program (Figure 13D). Its design was inspired by the animal and preliminary clinical research. X°Port has not yet received FDA-clearance or a CE-mark. The device was designed for donor lung storage at 10°C and is being utilized in a multicenter randomized trial with Northern American and European centers [43].

## Clinical Studies

### The Proof-Of-Concept for 10°C CHS in Lung Transplantation With the myTEMP 65HC Incubator

In 2021, the first clinical proof-of-concept study of donor lung CHS was reported by Toronto. In five bilateral LTx cases, extended 10°C CHS in the myTEMP 65HC incubator was performed to avoid overnight transplantation [9]. After procurement, donor lungs were first transported by SIS. In the transplant center, lungs were transferred to the incubator at 10°C. Total ischemic time was extended up to 16h30. No incidence of primary graft dysfunction grade 3 (PGD3) was observed at 72 h and all patients were reported to be alive at post-LTx day 330. This study was the first to show the possibility of safely bridging the night using CHS.

### Multicenter Prospective Non-randomized Trial of 10°C CHS With the myTEMP 65HC Incubator

In 2023, the results of a multicenter prospective non-randomized trial on 10°C CHS were published by Toronto, Vienna, Madrid and Florida [16]. CHS at 10°C in 70 LTx cases was compared to 140 propensity matched SIS cases. Donor lungs were transported with SIS to the transplant center and transferred to the incubator set at 10°C if cross-clamp time was expected between 6 PM and 4 AM. Recipient anesthesia was induced after 6 AM. Authors aimed to study if extended preservation with CHS at 10°C is safe, and to compare outcome with LTx after SIS.

Total ischemic time of the second implanted lung was extended to a median of 14h08. There was no significant difference for PGD3 at 72 h (5.7% for CHS vs. 9.3% for SIS), 30-day -and 1-year survival, showing that extending lung preservation time up to 12 h with 10°C CHS is safe.

### GUARDIAN-LUNG Registry for Post-market Registration of LUNGguard Utilization

The Global Utilization and Registry Database for Improved preservAtion of doNor LUNGs (GUARDIAN-LUNG) aims to collect real-world data on LTx with CHS in LUNGguard. In a preliminary analysis, 86 LUNGguard and 90 SIS cases were compared [44]. Incidence of PGD3 at 72 h was not significantly different but a trend with 54% reduction after LUNGguard preservation was observed. Kaplan-Meier analysis revealed a significantly improved survival for LUNGguard vs. SIS with a one-year survival rate of 92.7% vs. 82.2%, respectively.

Another preliminary study compared short-term outcome and costs after LTx with LUNGguard vs. SIS [45]. Total ischemic times were similar for both preservation techniques. The incidence of PGD3 at 72 h and rejection rate prior to discharge and survival were similar.

Interestingly, in 2023, results from the GUARDIAN-heart registry including 569 patients (255 SherpaPak vs. 314 ice) in heart transplantation were reported [46]. Data indicated a favorable outcome after CHS of donor hearts in SherpaPak with significantly lower incidence of PGD and a trend towards reduced need for post-transplant mechanical circulatory support and improved 1-year survival.

### Multicenter Randomized Controlled Trial With X°port

In 2023, a multicenter prospective clinical randomized controlled trial (RCT) was set up by Toronto comparing 10°C CHS in the X°Port vs. SIS [43]. The aim of this study is to show non-inferiority of extended preservation at 10°C in X°Port. End-points are PGD3 at 72 h, postoperative recovery, acute rejection, performance status and 1-year survival.

## Overnight Bridging of Lung Preservation With CHS

Conventionally, there has been an emphasis on restricting CIT to within the acceptable range of 6–8 h using traditional ice storage [13]. Nevertheless, there have been reports of successful LTx cases exceeding the 8 h timeframe, and retrospective studies revealed inconclusive correlation between ischemia time and post-LTx outcomes [47–49]. Furthermore, within the framework of SIS, the transplant team contends with persistent time constraints and a tendency for overnight transplantation. This situation presents logistical challenges, especially in the context of remote and distant procurements.

The effect of performing transplantation outside regular working hours has been studied for different organs, and available data show potential negative impact on outcome [50]. In a retrospective cohort of 563 LTx cases, a higher incidence of PGD3 was observed within 72 h when reperfusion was between 4 AM and 8 AM [51]. In a propensity matched study on LTx, 187 overnight cases were compared with 187 daytime cases. Overnight transplantation was associated with higher rates of postoperative major events, worse 5-year survival and shorter freedom from bronchiolitis obliterans syndrome [14].

Safely extending ischemic times with CHS can overcome the challenges associated with time pressure. Longer transport time allows procurement in hospitals located further away. Limited capacity at the operating theatre or multi-organ transplants can be managed more easily [52]. Shorter intervals between procurement allow consecutive LTx procedures. Hence, CHS introduces more flexibility for transplant teams without compromising outcome, which benefits healthcare personnel. Daytime transplantation improves overall fitness of transplant teams favoring cognitive and psychomotor skills and reducing the likelihood of errors. An anonymous survey of 7,900 correspondents reported an association of burnout and decreased career satisfaction with overnight surgery. A significantly higher proportion of surgeons working >80 h/week or >2 nights on call/week indicated that they would not become a surgeon again [53]. Furthermore, daytime LTx provides more technical expertise and personnel available. Also, providing a scheduled daytime LTx would attract young professionals and retain more surgeons and transplant personnel.

## LUNGguard Utilization and Experience at University Hospitals Leuven

CHS with LUNGguard was introduced in our center (Leuven, Belgium) in November 2022. Our current policy states that CHS with LUNGguard is used for cases with donor cross-clamp after 6 PM with recipient anesthesia starting at 7.30 AM, in cases of logistical limitations to accept valid donor offer, and if a complex recipient procedure is anticipated. By the end of February 2024, CHS with LUNGguard was performed in 24 cases. The average donor age was 60 years (12M/12F), 7 DCD cases were performed. In 18 (75%) cases, LUNGguard was used to extend preservation until daytime. The ischemic time of the second implanted lung exceeded 12 h in 18 (75%) and 15 h in 15 (63%) cases. The average preservation temperature was 6.8°C. Median total ischemia time for the second implanted lung was 15 h with a maximum of 22 h. The incidence of PGD3 at 72 h was 0% and median postoperative time on ventilator was 39 h. One patient died 1 week after LTx, suffering from the consequences of intraoperative ECMO-failure [54, 55].

## Leaving the Ice Age Behind: The Dawn of a New Era

Research encompassing historical and recent animal experiments, groundbreaking clinical studies and the development of new preservation devices have shown that CHS of donor lungs is here to stay. The ice box has served transplant teams for decades but an irreversible shift towards use of CHS has set off. Reaching donor hospitals located further away and extending preservation to daytime can increase the number of transplants and improve outcome.

Larger patient numbers and longer follow-up are needed to study the safety of extended CHS preservation with associated reduction of PGD and prolongation of survival. Currently, temperatures for CHS are between 6°C and 10°C, but optimal temperature for CHS has not yet been pinpointed. Another interesting subject is to investigate whether CHS in general or prolonged preservation with CHS improves outcome for extended criteria donor lungs (injury, pneumonia). More research on the optimal preservation duration, temperature and unraveling the mechanism of CHS remain topics for further research. Further investigation is also required to determine whether the advantages of CHS are solely dependent on the presence of oxygen in the alveoli, making them specific to the lung, or if they also apply to other solid organs.

## Conclusion

Based on preclinical and clinical data, we reviewed the underlying mechanisms of improved lung preservation with CHS. It was shown that preservation of lungs by CHS better maintains mitochondrial health and cellular viability. The current clinical data supports the feasibility of implementing CHS to safely extend preservation time, and to avoid overnight transplantation. Additional experimental, clinical studies and RCTs are necessary to further define the future of preservation in LTx. Furthermore, the potential benefits of CHS for other solid organs also require thorough investigation.

## Figures and Tables

**Table:** 

**AFC**	alveolar fluid clearance
**AsA**	ascorbate
**ATP**	adenosine triphosphate
**CE**	conformité Européenne
**CHS**	controlled hypothermic storage
**CIT**	cold ischemic time
**COPD**	chronic obstructive pulmonary disease
**CT**	core temperature
**DBD**	donation after brain death
**DCD**	donation after circulatory death
**ECMO**	extracorporeal membrane oxygenation
**ETC**	electron transport chain
**EVLP**	*ex-vivo* lung perfusion system
**FDA**	food and drug administration
**FiO**_**2**_	fraction of inspired oxygen
**G-6-P**	Glucose-6-phosphate
**GSH**	gluthathione
**GUARDIAN**	Global Utilization And Registry Database for Improved preservation of DoNor
**ICU**	intensive care unit
**IL-1**	interleukin-1
**IL-1β**	interleukin-1β
**IRI**	ischemia reperfusion injury
**ISHLT**	International Society for Heart and Lung Transplantation
**LDH**	lactate dehydrogenase
**LTx**	lung transplantation
**mtDNA**	mitochondrial desoxyribonucleic acid
**PaO2**	arterial pressure of Oxygen
**PAP**	pulmonary artery pressure
**PCr**	phosphocreatine
**PGD**	primary graft dysfunction
**PGD3**	primary graft dysfunction grade 3
**RCT**	randomized controlled trial
**RET**	reverse electron transport
**ROS**	reactive oxygen species
**SDH**	succinate dehydrogenase
**SIS**	static ice storage
**ST**	surface temperature
**Tx**	transplantation
**W/D**	wet-to-dry ratio
